# The Effect of the Area Deprivation Index on Surgical Outcomes for Benign Cystectomy

**DOI:** 10.1155/aiu/6140836

**Published:** 2026-07-22

**Authors:** Maxwell Sandberg, David Thole, Randall Bissette, Madeline Sandberg, Dylan Wolff, Lindsay Schwartz, Ethan Weitzman, Gregory Russell, Robert Evans, Alejandro Rodriguez

**Affiliations:** ^1^ Department of Urology, Wake Forest University School of Medicine, Winston Salem, North Carolina, USA, unc.edu; ^2^ Department of Psychology, University of Michigan-Ann Arbor, Ann Arbor, Michigan, USA

## Abstract

**Introduction:**

The impact of socioeconomic status (SES) on surgical outcomes is often underrecognized. Benign cystectomy (BC) for nononcologic bladder disease carries high morbidity but remains understudied. We evaluated the effect of SES on postoperative outcomes and healthcare utilization following BC.

**Methods:**

We retrospectively reviewed all BCs performed at our institution from 2012 to 2025. Neighborhood‐level socioeconomic disadvantage was measured using the area deprivation index (ADI; scale 1–10), with higher scores indicating greater deprivation. Patients were stratified into deciles (ADID). ADI reflects area‐level socioeconomic conditions rather than individual‐level characteristics and therefore may not capture patient‐specific socioeconomic factors. Surgical outcomes, emergency department (ED) utilization, and 90‐day readmissions were compared across ADID groups.

**Results:**

Among 183 patients, the median ADI was 5 (IQR 4–8). Preoperative comorbidities, urinary diversion type, operative time, length of stay, and in‐hospital complication rates were similar across ADID groups. ED visits and 90‐day readmission rates did not differ by SES (*p* > 0.05). On multivariable analysis, Indiana pouch diversion was associated with higher odds of postoperative complications (OR, 4.2; 95% CI, 1.4–12.8; *p* < 0.05). Both Indiana pouch (OR, 4.1; 95% CI, 1.3–13.1; *p* < 0.05) and a neobladder (OR, 3.7; 95% CI, 1.4–9.8; *p* < 0.05) were associated with a greater likelihood of readmission within 90 days of discharge.

**Conclusions:**

SES, as measured by ADID, was not significantly associated with outcomes or postoperative healthcare utilization. BC carries high morbidity regardless of SES. These findings warrant prospective validation.

## 1. Introduction

Socioeconomic status (SES) incorporates a person’s economic status, educational status, income, and living location. Traditional thought in urologic surgery focused on both modifiable and nonmodifiable risk factors and their impact on patient outcomes like body mass index, age, sex, race, and comorbidities. While undoubtedly important, patient SES and its impact on surgical outcomes were often overlooked. Over time, the relevance of SES became better understood. This includes studies showing different surgical selection options for stone treatment based on patient race and income, different surgical selection choices based on neighborhood SES, and earlier age at diagnosis of genitourinary cancers, as examples [1–3].

The area deprivation index (ADI) is a tool to map socioeconomic conditions of neighborhoods based on public data using income, education, employment, and housing quality [4, 5]. Scores are assigned using census block data on a scale of 1–10, with 1 being the least disadvantaged and 10 being the most socioeconomically disadvantaged. Additionally, national decile rankings are included, and higher rankings indicate greater disadvantage [4, 5]. Worse ADI scores (lower SES) have been associated with poor cancer‐specific survival for breast, colon, lung, and prostate cancer [6].

Benign cystectomy (BC) involves the removal of part or all of the bladder, plus or minus the removal of sexual organs like the uterus and prostate, for reasons not due to bladder cancer or other malignancy. Indications for BC include but are not limited to neurogenic bladder, end‐stage interstitial cystitis/bladder pain syndrome (IC/BPS), fistula, and radiation cystitis [7, 8]. BC is estimated to account for 5.4%–8% of all cystectomies performed across the United States [7–11]. Further, BC carries a significant complication rate, estimated at around 35%–50%, even greater than cystectomy for cancer [8, 12–14]. In the past, Knorr et al. have demonstrated higher patient mortality and worse oncologic outcomes in patients undergoing radical cystectomy (RC) for bladder cancer with greater ADI quartiles, meaning worse SES [15]. Yet, no such study has researched the relationship between ADI and BC outcomes.

The purpose of this study was to identify associations between perioperative outcomes, complications, emergency department (ED) utilization, and readmission after BC patients and SES using ADI scores.

## 2. Materials/Methods

**FIGURE 1:**
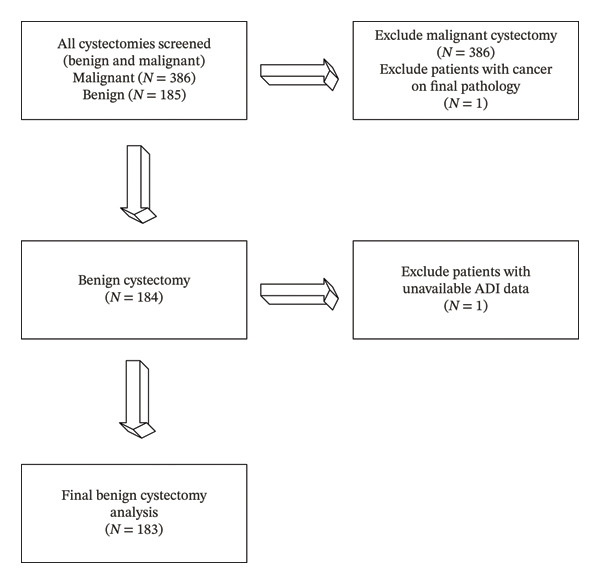
Patient flow diagram. This figure displays screening, exclusion, and selection criteria for patients in the study.

This was a retrospective single‐institutional study of all patients who underwent BC between 2012 and 2025 at our institution. All patients who underwent RC for bladder cancer were excluded (*N* = 386), as were patients who underwent BC, but whose final pathology revealed cancer (*N* = 1). A small portion of patients underwent RC but for benign indications, and these patients were still included in the final analysis (*N* = 3). Indications for BC were divided into neurogenic bladder, end‐stage IC/BPS, and “other reasons.” Patient flow selection for the study is detailed in Figure 1. Demographic information, including age at surgery, sex, race, patient comorbidities, and Charlson Comorbidity Index (CCI), was reviewed. Patient ADI score was recorded using U.S. Census block group data through publicly available mapping tools with national deciles from the 2023 Version of the ADI (most recent) [4, 5]. Patient addresses were self‐reported and geocoded by hospital employees into the electronic medical record at the time of patient check‐in for surgery and/or admission to the hospital. Patients with missing or unstable addresses were excluded from any ADI analysis in the study (*N* = 1). True individual SES was not measured. Perioperative/postoperative information, including bladder capacity prior to BC, operative time, length of stay, complication rate, complication type, ED utilization, and readmission rate, was tabulated. ED visits and readmissions were reviewed up to 90 days from discharge. Bladder capacities were determined by therapeutic hydrodistension for IC/BPS patients. All operative approaches to BC were open. Complication grades were calculated using the Clavien Dindo complication system [16]. Major complications were considered ≥ Clavien IIIa. Complications were divided into those that occurred in‐house immediately following BC and those occurring within 90 days of discharge from the hospital for BC requiring readmission to the hospital. Complications were also qualified according to the cystectomy system as reported by Shabsigh et al., and patients could have multiple complications postoperatively [17]. Discharge destination was either home, home with nursing assistance, or skilled nursing facility. The most recent urologic follow‐up was recorded.

Patients were categorized into four groups based on collapsed ADI deciles (ADID 1‐2, 3–5, 6–8, and 9–10). These groupings were selected to maintain consistency with prior ADI‐based surgical literature and to reflect clinically meaningful strata of socioeconomic disadvantage [15, 18, 19]. Continuous variables were assessed for normality using Komolgorov–Smirnov testing, and Supporting Table 1 provides results for this testing. Normally distributed continuous variables were compared using analysis of variance (ANOVA), and nonnormally distributed continuous variables were compared using Kruskall–Wallis *H* test. Categorical variables were compared using chi‐squared (gender, patient comorbidities, anastomosis type, complication rate, discharge destination, ED visitation, readmission, and stricture development) and Fischer’s exact (race, BC indication, complication type, Clavien grade, and urinary diversion) testing. Forward binary logistic regression modeling was performed to predict the likelihood of in‐house complication rates based on ADID and readmission to the hospital within 90 days of discharge from BC. The model included variables with*p* < 0.1 on univariable analysis and/or variables deemed of high clinical relevance. An event rate of 10 per variable was utilized for the regression models. No collinearity between variables in the models existed, and the model was not overfit based on events per variable. Patients with missing data points on variables (*N* = 1) in the model were excluded from inclusion for that particular regression model, as were patients with percutaneous nephrostomy tubes (PCNs; *N* = 1) as their urinary diversion. Kaplan–Meier analysis with log‐rank test was performed to assess differences in 90‐day readmission rates by ADID.

Sensitivity modeling, using ADI as both a continuous and ordinal variable, was also performed to assess the direction and magnitude of effect estimates.

## 3. Results

A total of 183 patients were included in the final analysis. There were 46 (25%) patients in D1 of ADI, 50 (27%) patients in D2, 57 (31%) patients in D3, and 30 (16%) patients in D4 (Table 1). There were 33 (72%) females in D1, 36 (72%) females in D2, 36 (63%) females in D3, and 21 (70%) females in D4 (*p* > 0.05). Race was not significantly different based on ADID. End‐stage IC/BPS was the main indication for BC in D1 (78%), D2 (80%), D3 (63%), and D4 (60%), with no significant differences in indication for BC by ADID. The most common urinary diversion type was an ileal conduit, followed by neobladder, and then an Indiana pouch, which were not significantly different in use by ADID. Bricker and Wallace ureteral anastomosis types were also not significantly different by ADID. Mean age at surgery was 61 years old in D1, 58 years old in D2, 56 years old in D3, and 54 years old in D4 (*p* > 0.05). On Kruskal–Wallis test, median CCI score (*H* = 1.3; df = 3), median bladder capacity at BC (*H* = 1.4; df = 3), median operative time (*H* = 3; df = 3), median length of stay (*H* = 0.9; df = 3), and median follow‐up (*H* = 7.6; df = 3) were not significantly different by ADID (*p* > 0.05). Rate of in‐house postoperative complications was 44% in D1, 50% in D2, 49% in D3, and 63% in D4, which was not statistically significantly different. A total of 8 (40%) in‐house complications were considered major in D1, 8 (32%) considered major in D2, 11 (38%) considered major in D3, and 4 (21%; *p* > 0.05) considered major in D4. The common complications experienced in the study were gastrointestinal (*N* = 38), bleeding (*N* = 26), and infectious (*N* = 12), which were not significantly different by ADID (*p* > 0.05). Discharge destination was not different by ADID.

**TABLE 1:** ADID comparisons.

Variable	DD1	D2	D3	D4	*p* value	*H*	Lower	Upper
*N*	46 (25)	50 (27)	57 (31)	30 (16)	—	—	—	—
Female	33 (72)	36 (72)	36 (63)	21 (70)	0.732	—	—	—
Age (years)	61 (15)	58 (16)	56 (17)	53 (15)	0.139	—	55	60
Reason for BC								
Neurogenic	3 (7)	4 (8)	5 (9)	6 (20)	0.158	—		
IC/BPS	36 (78)	40 (80)	36 (63)	18 (60)				
Other reasons	7 (15)	6 (12)	16 (28)	6 (20)				
Race								
Caucasian	40 (87)	46 (92)	47 (81)	27 (90)	0.976	—		
Black	3 (7)	2 (4)	5 (9)	3 (10)				
Hispanic	1 (2)	1 (2)	2 (4)	0				
Asian	1 (2)	0	1 (2)	0				
Native American	0	1 (2)	1 (2)	0				
Pacific Islander	1 (2)	0	0	0				
Other	0	0	1 (2)	0				
CCI	3 (2–5)	3 (2–4.25)	3 (2–5)	3 (1–4)	0.759	1	3	4
Diabetes mellitus	9 (20)	17 (34)	14 (35)	6 (20)	0.351	—	—	—
Hypertension	19 (41)	23 (46)	31 (54)	14 (47)	0.605	—	—	—
Coronary artery disease	5 (11)	11 (22)	14 (25)	5 (17)	0.321	—	—	—
COPD	1 (2)	1 (2)	4 (7)	2 (7)	0.469	—	—	—
Bladder capacity (mL)	275 (200–437.5)	300 (175–437.5)	300 (212.5–437.5)	300 (225–425)	0.702	1	284	364
Operative time (minutes)	368 (293.5–399.5)	337 (265.5–387)	338 (282–385)	390 (328–450)	0.086	7	335	360
Urinary diversion								
Ileal conduit	35 (76)	39 (78)	44 (77)	22 (73)	0.397	—		
Neobladder	6 (13)	4 (8)	3 (5)	7 (24)				
Indiana pouch	5 (11)	7 (14)	9 (16)	1 (3)				
PCNs	0	0	1 (2)	0				
Bricker	2/35 (6)	6/39 (15)	7/44 (16)	2/22 (9)	0.444	—		
Wallace	33/35 (94)	33/39 (85)	37/44 (84)	20/22 (91)				
Stents	45 (98)	47 (94)	56 (98)	30 (100)	0.375	—	—	—
In‐house complication	20 (44)	25 (50)	28 (49)	19 (63)	0.443	—	—	—
Clavien grade in‐house								
I	3 (15)	6 (24)	6 (21)	0	0.244	—		
II	9 (45)	11 (44)	12 (41)	15 (80)				
IIIa	4 (20)	3 (12)	5 (17)	2 (10)				
IIIb	1 (5)	0	1 (3)	0				
IVa	1 (5)	5 (20)	2 (7)	2 (10)				
IVb	2 (10)	0	2 (7)	0				
V	0	0	1 (3)	0				
Complication type								
Infectious	0	6 (24)	4 (13)	2 (9)	0.404	—		
Bleeding	5 (19)	4 (17)	11 (35)	6 (27)				
Surgical	1 (4)	0	0	1 (5)				
Wound	3 (11)	2 (8)	2 (6)	1 (5)				
Genitourinary	0	1 (4)	1 (3)	0				
Gastrointestinal	11 (41)	7 (29)	10 (31)	10 (44)				
Thromboembolic	0	1 (2)	0	0				
Cardiac	0	0	0	0				
Pulmonary	2 (7)	2 (8)	1 (3)	1 (5)				
Neurologic	0	0	1 (3)	0				
Miscellaneous	3 (11)	0	2 (6)	1 (5)				
Other	2 (7)	2 (8)	0	0				
Length of stay (days)	6 (5–8)	5 (5–7)	6 (5–7)	7 (4–8)	0.825	1	6	8
Discharge destination								
Home	20 (43)	28 (56)	23 (40)	18 (60)	0.294	—		
Home with nursing assistance	22 (48)	18 (36)	30 (53)	8 (27)				
Skilled nursing facility	4 (9)	4 (8)	4 (7)	4 (13)				
ED visit postoperatively	29 (63)	21 (43)	27 (49)	14 (47)	0.235	—	—	—
Number of ED visits postoperatively	1 (1–2)	2 (1–2)	1 (1–2)	1 (1–4)	0.454	3	1	2
Readmission within 90 days	18 (39)	18 (38)	24 (45)	9 (33)	0.756	—	—	—
Clavien grade readmission								
I	6 (21)	2 (10)	2 (8)	0	0.233	—		
II	15 (53)	9 (43)	10 (38)	4 (45)				
IIIa	4 (14)	3 (14)	6 (23)	0				
IIIb	1 (4)	3 (14)	7 (27)	2 (22)				
IVa	1 (4)	3 (14)	0	1 (11)				
IVb	1 (4)	1 (5)	0	1 (11)				
V	0	0	1 (4)	1 (11)				
Ureteroenteric stricture postoperatively	8 (17)	7 (14)	10 (18)	9 (30)	0.36	—	—	—
Follow‐up (months)	43 (9–83)	19 (6–53)	18.5 (4–55)	43 (13–88)	0.053	8	33	44

*Note:* This table compares patients by ADID on a variety of variables in the study cohort. Each group is represented. Continuous variables are medians with 25th and 75th percentiles in parentheses, aside from age, which is the mean with standard deviation. Categorical variables are totals with the percentage of the cohort in parentheses. H represents the test statistic for the Kruskal–Wallis test on nonnormally distributed continuous variables. Confidence intervals for group differences are provided where relevant.

A total of 29 (63%) patients visited the ED after discharge from BC in D1, 21 (43%) in D2, 27 (49%) in D3, and 14 (47%) in D4, which was not statistically significantly different. On Kruskal–Wallis test, the median number of ED visits postoperatively by ADID was also not statistically significantly different (*H* = 2.6; df = 3). There were 18 (39%) patients readmitted to the hospital within 90 days of discharge in D1, 18 (38%) patients readmitted in D2, 24 (45%) patients readmitted in D3, and 9 (33%; *p* > 0.05) patients readmitted in D4. Rate of major complications requiring readmission were 25% in D1, 48% in D2, 54% in D3, and 50% in D4 (*p* > 0.05). Ureteral stents postoperatively were used in 98% of D1 patients, 94% of D2, 98% of D3, and 100% of D4, which did not differ. Development of ureteroenteric stricture postoperatively from BC was 17% in D1, 14% in D2, 18% in D3, and 30% in D4, which was not significantly different. A total of 6 (13%) patients died in D1, 5 (10%) died in D2, 11 (19%) died in D3, and 4 (13%) died in D4, with no significant difference in prevalence of death by ADIQ.

**FIGURE 2:**
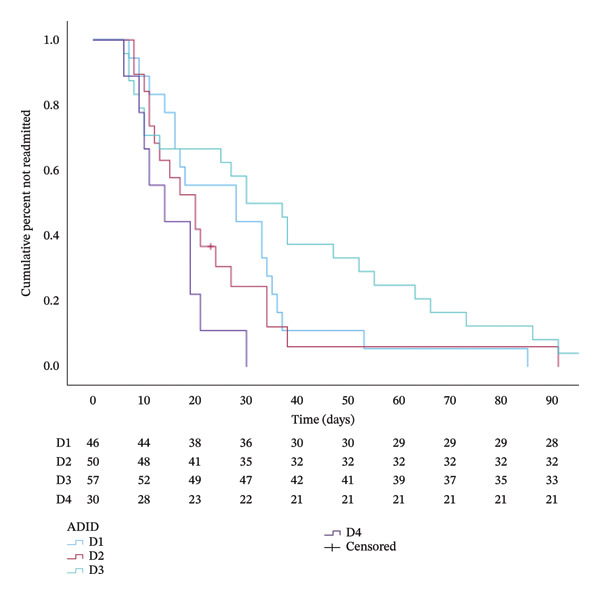
Kaplan–Meier analysis. This figure displays readmission after BC within 90 days of discharge by ADID in the study analysis. The proportion not readmitted is on the *y*‐axis, and time is on the *x*‐axis in days. No patients died prior to the conclusion of the 90‐day readmission window. Log‐rank test of significance value is also provided with a number at risk table below the figure.

On multivariable logistic regression (Table 2), when controlling for sex, CCI, surgical indication, and urinary diversion, ADID was not associated with the likelihood of developing an in‐house complication (*p* > 0.05). An Indiana pouch was associated with a greater likelihood of incurring a postoperative complication after BC (odds ratio [OR], 4; confidence interval [CI] 2–12; *p* < 0.05]. On multivariable logistic regression (Table 3), when controlling for sex, CCI, surgical indication, and urinary diversion, ADID was not associated with the likelihood of 90‐day readmission to the hospital. Both an Indiana pouch (OR 4; CI 1, 13; *p* < 0.05) and a neobladder (OR, 4; CI, 1–10; *p* < 0.05) were associated with a greater likelihood of readmission within 90 days of discharge. On log‐rank test for Kaplan–Meier survival analysis when comparing 90‐day readmission after BC by ADID, no significant difference was appreciated (Figure 2; *p* > 0.05).

**TABLE 2:** ADID multivariable regression model.

Variable	Significance	Odds ratio (OR)	OR 95% CI
Sex (male vs. female)	0.806	1	0.5–2
CCI (per 1)	0.046	1	1–2
ADID (overall)	0.568	—	—
D1	0.182	0.5	0.2–1
D2	0.477	0.7	0.3–2
D3	0.266	0.6	0.2–2
D4 (reference)	—	—	—
BC indication (overall)	0.206		
Neurogenic bladder vs. IC/BPS	0.124	2	0.8–7
Other vs. IC/BPS	0.233	2	0.7–4
IC/BSP (reference)	—	—	—
Urinary diversion type (overall)	0.019		
Indiana pouch vs. ileal conduit	0.0055	4	2–12
Neobladder vs. ileal conduit	0.244	2	0.6–8
Ileal conduit (reference)	—	—	—

*Note:* This table shows a regression model with an in‐house complication after BC as the outcome, with 182 patients included. Significance is the *p* value, OR is the adjusted odds ratio, and 95% CI is the confidence intervals for each variable. Any variables not displayed in the model can be assumed not to carry statistical significance or to be collinear with other model variables. For ADID, D4 is the referent group; for urinary diversion, the ileal conduit is the referent group; and for the indication, “IC/BPS” is the referent group.

**TABLE 3:** ADID multivariable regression model.

Variable	Significance	Odds ratio (OR)	OR 95% CI
Sex (male vs. female)	0.822	1	0.4–2
CCI (per 1 unit)	0.777	1	0.9–1
ADIQ (overall)	0.567	—	—
D1	0.404	2	0.5–5
D2	0.451	2	0.5–4
D3	0.161	2	0.8–6
D4 (reference)	—	—	—
Bladder cancer indication (overall)	0.368		
Neurogenic bladder vs. IC/BPS	0.179	2	0.7–6
Other vs. IC/BPS	0.454	1	0.6–3
IC/BSP (reference)	—	—	—
Urinary diversion type (overall)	0.007		
Indiana pouch vs. ileal conduit	0.010	4	1–10
Neobladder vs. ileal conduit	0.017	4	1–13
Ileal conduit (reference)	—	—	—

*Note:* This table shows a regression model with 90‐day readmissions after BC as the outcome, with 182 patients included. B is the unadjusted odds ratio, S.E. is the standard error, significance is the *p* value, Exp (B) is the adjusted odds ratio, and 95% CI is the confidence intervals for each variable. Any variables not displayed in the model can be assumed not to carry statistical significance or to be collinear with other model variables. For ADID, D4 is the referent group; for urinary diversion, the ileal conduit is the referent group; and for the indication, “IC/BPS” is the referent group.

Sensitivity analyses modeling ADI as both a continuous and ordinal variable yielded consistent results, with ADI remaining nonsignificant and no meaningful changes in the direction or magnitude of effect estimates. These findings support the robustness of the primary model. In the complication model, the *p* value using ADID is 0.57; if ADI is modeled continuously, the *p* value becomes 0.23, and if the categories are modeled ordinally, the *p* value is 0.30. Similarly, in the readmission model, the categorized ADID *p* value is 0.57, the continuous *p* value is 0.58, and the ordinal *p* value is 0.75.

## 4. Discussion

This large retrospective analysis of BC attempted to identify the impact of SES on peri/postoperative outcomes by using ADI scores and is the newest paper after updating our data in a series of BC that our research group has published previously. No relevant associations were identified based on ADID. This contrasts with RC, where previous research on ADI found worse outcomes with higher ADI scores [15]. Knorr et al. noted greater rates of clinical and pathologic muscle‐invasive bladder cancer, pathologic node‐positive bladder cancer, and variant histology in D1 compared to D4 [15]. Additionally, worse ADID was associated with greater 30‐day and 90‐day mortality rates. Golombos et al. compared RC patients with bladder cancer using patient home zip codes as a proxy for SES, noting higher perioperative complications with worse SES [20]. On Kaplan–Meier analysis, no differences in overall survival after BC were identified by ADI score. It is important to recognize that BC patients are uniquely different than those with bladder cancer undergoing RC, both in terms of demographics and outcomes, as demonstrated in multiple comparative studies [8, 21]. Moreover, in the past, our research group has demonstrated better SES (lower ADI scores) in BC patients relative to those with bladder cancer who underwent RC [8]. There are likely other factors aside from SES that account for the lack of significant differences identified in this study compared to prior studies on RC patients with bladder cancer.

Despite no significant differences in outcomes by ADID, several observational findings are worth discussion from the BC cohort assessed. No difference in urinary diversion selection was appreciated, and there was a high proportion of continent cutaneous diversions overall, making up 13% of urinary diversions in D1, 8% in D2, 16% in D3, and 23% in D4. On regression analysis, an Indiana pouch was associated with a greater likelihood of a postoperative complication, and both an Indiana pouch and a neobladder were associated with readmission to the hospital. A previous study by Maurice et al. found that higher patient income predicted a greater likelihood of a continent cutaneous diversion or a neobladder for those undergoing RC for bladder cancer [22]. Kim et al. found that patients insured by Medicaid were also less likely to receive a continent urinary diversion [23]. To our knowledge, no study has previously assessed urinary diversion in BC and SES, but these findings contradict studies performed on RC for bladder cancer. While no significant differences in complication rate were appreciated, the overall rate of in‐house complications, and high‐grade in‐house complications were high, irrespective of ADID, which fits with established literature [10, 24]. Fifty percent of patients had at least one ED visit after BC, and while no significant differences were noted, D1 had the highest utilization rate. Likewise, readmission rates within 90 days of BC were also not statistically significantly different amongst all ADIDs. Interestingly, many publications show greater healthcare usage after surgery to be linked to worse SES, yet for BC, our data indicate resource use was high across all ADI groups [25, 26]. Furthermore, discharge destination had no association with ADID, fitting with prior studies on RC for cancer [27]. In total, these observations seem to demonstrate that previously established associations between low SES and outcomes for RC in bladder cancer do not necessarily translate to BC patients.

There are multiple limitations in this study that require identification. First, this is a retrospective review and is subject to the inherent limitations that come with medical record chart reviews, like selection bias and uncertainty regarding documentation. Additionally, a single‐center analysis limits the external validity of the study findings. While utilizing ADI as a measure of SES has been validated, it is not the best measurement of an individual’s SES and is better for group SES, and this paper opens itself up to the possibility of ecological fallacy. The ADI values utilized for this publication are from the most recent 2023 Version, and we cannot be certain how long a patient lived in a certain region, nor if the ADI value associated with this location changed during the study window, which is a limitation due to the retrospective design of this study. Furthermore, our cohort was large for BC, which is a relatively uncommon procedure, but 183 patients are still a small sample size to analyze. All procedures were performed in an open fashion based on the surgeons’ comfort in this study, and it is possible that this influenced complication rates/the types of complications patients experienced. The regression models would be improved by including calendar year, surgeon, indication severity, insurance status, distance from hospital, rurality, race and ethnicity, smoking, body mass index, functional status, frailty, prior radiation, prior pelvic surgery, chronic infection, chronic pain diagnosis, and preoperative opioid use if available. Unfortunately, due to a significant portion of missing variables/unavailable information in the patient record on the cohort studied, we elected to exclude these variables from the models. We also did not have equal proportions of patients in each ADID; specifically, D4 had the least number of patients. With a greater share of D4, it is possible results could have differed, particularly if patients of the lowest SES drive potential differences. Regarding statistical power, the present study may be underpowered to detect modest but clinically meaningful differences between ADI groups. For example, observed differences in complication rates across groups may be clinically relevant despite not reaching statistical significance, and confidence intervals were wide, reflecting imprecision in these estimates. Lastly, this paper comes after a prior manuscript that our research group has published on SES after cystectomy and uses a similar patient dataset [8]. However, the prior focus was comparing BC to RC patients only, and the goal was to assess differences in patients undergoing cystectomy for benign versus oncologic reasons. After updates to the patient dataset, we felt an expanded analysis with BC patients alone, and an emphasis on SES was warranted, given the paucity of data in the literature on this topic in a unique patient cohort.

## 5. Conclusion

BC carries a high morbidity rate postoperatively, and healthcare utilization is high after surgery. This pattern held true, irrespective of patient ADID. These results indicate that no association was detected with SES and BC. Nevertheless, this study is exploratory and hypothesis‐generating in nature and should be viewed in the context of its multiple limitations. These results call for validation, which would entail assigning ADI scores at the time of BC to all patients and prospectively following them postoperatively for an extended period, ensuring to record any address changes and if this impacted the previously assigned ADI score.

## Funding

The authors have nothing to report.

## Ethics Statement

This study was approved by the Wake Forest University School of Medicine Institutional Review Board IRB00114519.

## Consent

Informed consent was waived by the Wake Forest University School of Medicine Institutional Review Board.

## Conflicts of Interest

The authors declare no conflicts of interest.

## Supporting Information

Additional supporting information can be found online in the Supporting Information section.

## Supporting information


**Supporting Information 1** Supporting Table 1. Komolgorov–Smirnov test of normality. This table shows results for tests of normality of continuous variables in the study. *p* values < 0.05 are nonnormally distributed and *p* values > 0.05 are normally distributed.

**Supporting Information 2** STROBE Statement—checklist of items that should be included in reports of observational studies.

## Data Availability

The data utilized in the publication of this manuscript are not publicly available due to patient privacy concerns but are available in a limited deidentified format upon reasonable request to the corresponding author.
