# Supplementary material for: The Effect of the Area Deprivation Index on Surgical Outcomes for Benign Cystectomy
Source: Adv Urol. 2026 Jul 22;2026:6140836. doi: 10.1155/aiu/6140836 (PMC13392508; doi:10.1155/aiu/6140836)
Supplement: Supplementary file 1 — Supporting Information 1 Supporting Table 1. Komolgorov–Smirnov test of normality. This table shows results for tests of normality of continuous variables in the study. p values < 0.05 are nonnormally distributed and p values > 0.05 are normally distributed. [file AIU-2026-6140836-s002.docx]

**Supplementary table 1. Komolgorov-Smirnov test of normality.** The following table shows results for tests of normality of continuous variables in the study. P-values > 0.05 indicate normally distributed variables.

| **Variable** | **Statistic** | **Significance** |
| --- | --- | --- |
| Age (years) | 0.101 | 0.200 |
| Bladder capacity (mL) | 0.178 | 0.003 |
| CCI | 0.202 | <0.001 |
| Operative time (minutes) | 0.151 | 0.026 |
| Length of stay (days) | 0.312 | <0.001 |
| Number of ED visits postoperatively | 0.310 | <0.001 |
| Follow-up (months) | 0.205 | <0.001 |
