# Supplementary material for: The Effect of the Area Deprivation Index on Surgical Outcomes for Benign Cystectomy
Source: Adv Urol. 2026 Jul 22;2026:6140836. doi: 10.1155/aiu/6140836 (PMC13392508; doi:10.1155/aiu/6140836)
Supplement: Supplementary file 2 — Supporting Information 2 STROBE Statement—checklist of items that should be included in reports of observational studies. [file AIU-2026-6140836-s001.docx]

STROBE Statement—checklist of items that should be included in reports of observational studies

|  | Item No. | Recommendation | Page  No. | Relevant text from manuscript |
| --- | --- | --- | --- | --- |
| **Title and abstract** | 1 | (*a*) Indicate the study’s design with a commonly used term in the title or the abstract | 2 | We retrospectively reviewed all BCs performed at our institution from 2012–2025. |
|  |  | (*b*) Provide in the abstract an informative and balanced summary of what was done and what was found | 2 | We retrospectively reviewed all BCs performed at our institution from 2012–2025. Neighborhood-level socioeconomic disadvantage was measured using the Area Deprivation Index (ADI; scale 1–10), with higher scores indicating greater deprivation. Patients were stratified into deciles (ADID). ADI reflects area-level socioeconomic conditions rather than individual-level characteristics and therefore may not capture patient-specific socioeconomic factors. Surgical outcomes, emergency department (ED) utilization, and 90-day readmissions were compared across ADID groups.  Among 183 patients, the median ADI was 5 (IQR 4–8). Preoperative comorbidities, urinary diversion type, operative time, length of stay, and in-hospital complication rates were similar across ADID groups. ED visits and 90-day readmission rates did not differ by SES (p>0.05). On multivariable analysis, Indiana pouch diversion was associated with higher odds of postoperative complications (OR 4.2, 95% CI 1.4–12.8; p<0.05). Both Indiana Pouch (Odds ratio 4.1; CI 1.3, 13.1; p<0.05) and a neobladder (Odds ratio 3.7; CI 1.4, 9.8; p<0.05) were associated with a greater likelihood of readmission within 90-days of discharge. |
| Introduction | | | |  |
| Background/rationale | 2 | Explain the scientific background and rationale for the investigation being reported | 3 | Yet, no such study has researched the relationship between ADI and BC outcomes. The purpose of this study was to identify associations between perioperative outcomes, complications, emergency department (ED) utilization, and readmission after BC patients and SES using ADI scores. |
| Objectives | 3 | State specific objectives, including any prespecified hypotheses | 3 | The purpose of this study was to identify associations between perioperative outcomes, complications, emergency department (ED) utilization, and readmission after BC patients and SES using ADI scores. |
| Methods | | | |  |
| Study design | 4 | Present key elements of study design early in the paper | 4 | This was a retrospective single institutional study of all patients who underwent BC between 2012-2025 at our institution. |
| Setting | 5 | Describe the setting, locations, and relevant dates, including periods of recruitment, exposure, follow-up, and data collection | 4 | This was a retrospective single institutional study of all patients who underwent BC between 2012-2025 at our institution. All patients that underwent RC for bladder cancer were excluded (N=386) as were patients that underwent BC, but whose final pathology revealed cancer (N=1). |
| Participants | 6 | (*a*) *Cohort study*—Give the eligibility criteria, and the sources and methods of selection of participants. Describe methods of follow-up  *Case-control study*—Give the eligibility criteria, and the sources and methods of case ascertainment and control selection. Give the rationale for the choice of cases and controls  *Cross-sectional study*—Give the eligibility criteria, and the sources and methods of selection of participants | 4 | This was a retrospective single institutional study of all patients who underwent BC between 2012-2025 at our institution. All patients that underwent RC for bladder cancer were excluded (N=386) as were patients that underwent BC, but whose final pathology revealed cancer (N=1). A small portion of patients underwent RC but for benign indications, and these patients were still included in the final analysis (N=3). Most recent urologic follow-up was recorded. |
|  |  | (*b*) *Cohort study*—For matched studies, give matching criteria and number of exposed and unexposed  *Case-control study*—For matched studies, give matching criteria and the number of controls per case |  |  |
| Variables | 7 | Clearly define all outcomes, exposures, predictors, potential confounders, and effect modifiers. Give diagnostic criteria, if applicable | 4-5 | All patients that underwent RC for bladder cancer were excluded (N=386) as were patients that underwent BC, but whose final pathology revealed cancer (N=1). A small portion of patients underwent RC but for benign indications, and these patients were still included in the final analysis (N=3). Indications for BC were divided into neurogenic bladder, end-stage IC/BPS, and “other reasons.” Patients were categorized into four groups based on collapsed ADI deciles (ADID 1–2, 3–5, 6–8, and 9–10). These groupings were selected to maintain consistency with prior ADI-based surgical literature and to reflect clinically meaningful strata of socioeconomic disadvantage.^15,18,19^ |
| Data sources/ measurement | 8* | For each variable of interest, give sources of data and details of methods of assessment (measurement). Describe comparability of assessment methods if there is more than one group | 4-5 | Patients were categorized into four groups based on collapsed ADI deciles (ADID 1–2, 3–5, 6–8, and 9–10). These groupings were selected to maintain consistency with prior ADI-based surgical literature and to reflect clinically meaningful strata of socioeconomic disadvantage.^15,18,19^ Continuous variables were assessed for normality using Komolgorov-Smirnov testing, and Supplementary table 1 provides results for this testing. Normally distributed continuous variables were compared using analysis of variance (ANOVA) and non-normally distributed continuous variables were compared using Kruskall-Wallis H test. Categorical variables were compared using chi-squared (gender, patient comorbidities, anastomosis type, complication rate, discharge destination, ED visitation, readmission, stricture development) and Fischer’s exact (race, BC indication, complication type, Clavien grade, urinary diversion) testing. Forward binary logistic regression modeling was performed to predict likelihood of in-house complication rates based on ADID and readmission to the hospital within 90-days of discharge from BC. The model included variables with p<0.1 on univariable analysis and/or variables deemed of high clinical relevance. |
| Bias | 9 | Describe any efforts to address potential sources of bias 7-8 |  | There are multiple limitations in this study that require identification. First, this is a retrospective review, and subject to the inherent limitations that come with medical record chart reviews like selection bias and uncertainty regarding documentation. Additionally, a single-center analysis limits the external validity of the study findings. While utilizing ADI as a measure of SES has been validated, it is not the best measurement of an individual’s SES and is better for group SES, and this paper opens itself up to the possibility of ecological fallacy. Furthermore, our cohort was large for BC, which is a relatively uncommon procedure, but 183 patients are still a small sample size to analyze. Regarding statistical power, the present study may be underpowered to detect modest but clinically meaningful differences between ADI groups. For example, observed differences in complication rates across groups may be clinically relevant despite not reaching statistical significance, and confidence intervals were wide, reflecting imprecision in these estimates. |
| Study size | 10 | Explain how the study size was arrived at | 4 | This was a retrospective single institutional study of all patients who underwent BC between 2012-2025 at our institution. All patients that underwent RC for bladder cancer were excluded (N=386) as were patients that underwent BC, but whose final pathology revealed cancer (N=1). A small portion of patients underwent RC but for benign indications, and these patients were still included in the final analysis (N=3). |

Continued on next page

| Quantitative variables | 11 | Explain how quantitative variables were handled in the analyses. If applicable, describe which groupings were chosen and why |  | 4 Continuous variables were assessed for normality using Komolgorov-Smirnov testing, and Supplementary table 1 provides results for this testing. Normally distributed continuous variables were compared using analysis of variance (ANOVA) and non-normally distributed continuous variables were compared using Kruskall-Wallis H test. |
| --- | --- | --- | --- | --- |
| Statistical methods | 12 | (*a*) Describe all statistical methods, including those used to control for confounding | 4-5 | Continuous variables were assessed for normality using Komolgorov-Smirnov testing, and Supplementary table 1 provides results for this testing. Normally distributed continuous variables were compared using analysis of variance (ANOVA) and non-normally distributed continuous variables were compared using Kruskall-Wallis H test. Categorical variables were compared using chi-squared (gender, patient comorbidities, anastomosis type, complication rate, discharge destination, ED visitation, readmission, stricture development) and Fischer’s exact (race, BC indication, complication type, Clavien grade, urinary diversion) testing. Forward binary logistic regression modeling was performed to predict likelihood of in-house complication rates based on ADID and readmission to the hospital within 90-days of discharge from BC. The model included variables with p<0.1 on univariable analysis and/or variables deemed of high clinical relevance. An event rate of ten per variable was utilized for the regression models. No collinearity between variables in the models existed and the model was not overfit based on events per-variable. Patients with missing data points on variables in the model were excluded from inclusion for that particular regression model as were patients with percutaneous nephrostomy tubes (PCNs; N=1) as their urinary diversion. Kaplan-Meier analysis with log-rank test was performed to assess differences in 90-day readmission rates by ADID. |
|  |  | (*b*) Describe any methods used to examine subgroups and interactions | 4-5 | Patients were categorized into four groups based on collapsed ADI deciles (ADID 1–2, 3–5, 6–8, and 9–10). |
|  |  | (*c*) Explain how missing data were addressed | 5 | Patients with missing data points on variables in the model were excluded from inclusion for that particular regression model as were patients with percutaneous nephrostomy tubes (PCNs; N=1) as their urinary diversion. |
|  |  | (*d*) *Cohort study*—If applicable, explain how loss to follow-up was addressed  *Case-control study*—If applicable, explain how matching of cases and controls was addressed  *Cross-sectional study*—If applicable, describe analytical methods taking account of sampling strategy |  |  |
|  |  | (*e*) Describe any sensitivity analyses | 5-6 | Sensitivity modeling, using ADI as both a continuous and ordinal variable was also performed to assess direction and magnitude of effect estimates.  Sensitivity analyses modeling ADI as both a continuous and ordinal variable yielded consistent results, with ADI remaining non-significant and no meaningful changes in the direction or magnitude of effect estimates. These findings support the robustness of the primary model.  In the complication model, the p-value using ADID is 0.57; if ADI is modeled continuously, the p-value becomes 0.23, and, if the categories are modeled ordinally, the p-value is 0.30.  Similarly, in the readmission model, the categorized ADID p-value is 0.57, the continuous p-value is 0.58, and the ordinal p-value is 0.75. |
| Results | | | | |
| Participants | 13* | (a) Report numbers of individuals at each stage of study—eg numbers potentially eligible, examined for eligibility, confirmed eligible, included in the study, completing follow-up, and analysed | 4-5 | All patients that underwent RC for bladder cancer were excluded (N=386) as were patients that underwent BC, but whose final pathology revealed cancer (N=1). A small portion of patients underwent RC but for benign indications, and these patients were still included in the final analysis (N=3). A total of 183 patients were included in the final analysis. There were 46 (25%) in D1 of ADI, 50 (27%) patients in D2, 57 (31%) patients in D3, and 30 (16%) patients in D4. There were 33 (72%) females in D1, 36 (72%) females in D2, 36 (63%) females in D3, and 21 (70%) females in D4 (p>0.05). |
|  |  | (b) Give reasons for non-participation at each stage 4 |  | All patients that underwent RC for bladder cancer were excluded (N=386) as were patients that underwent BC, but whose final pathology revealed cancer (N=1). A small portion of patients underwent RC but for benign indications, and these patients were still included in the final analysis (N=3). |
|  |  | (c) Consider use of a flow diagram | 18 | Figure 1. Patient flow diagram. The following figure displays screening, exclusion, and selection criteria for patients in the study. |
| Descriptive data | 14* | (a) Give characteristics of study participants (eg demographic, clinical, social) and information on exposures and potential confounders | 5, 13 | A total of 183 patients were included in the final analysis. There were 46 (25%) in D1 of ADI, 50 (27%) patients in D2, 57 (31%) patients in D3, and 30 (16%) patients in D4. There were 33 (72%) females in D1, 36 (72%) females in D2, 36 (63%) females in D3, and 21 (70%) females in D4 (p>0.05).  **Table 1. ADID comparisons.** The following table compares patients by ADID on a variety of variables in the study cohort. Each group is represented. Continuous variables are medians with 25^th^ and 75^th^ percentiles in parentheses aside from age, which is mean with standard deviation. Categorical variables are totals with percentage of the cohort in parentheses. H represents the test statistic for the Kruskal-Wallis test on non-normally distributed continuous variables. Confidence intervals for group differences are provided where relevant. |
|  |  | (b) Indicate number of participants with missing data for each variable of interest | 13 | **Table 1. ADID comparisons.** The following table compares patients by ADID on a variety of variables in the study cohort. Each group is represented. Continuous variables are medians with 25^th^ and 75^th^ percentiles in parentheses aside from age, which is mean with standard deviation. Categorical variables are totals with percentage of the cohort in parentheses. H represents the test statistic for the Kruskal-Wallis test on non-normally distributed continuous variables. Confidence intervals for group differences are provided where relevant. |
|  |  | (c) *Cohort study*—Summarise follow-up time (eg, average and total amount) | 5, 13 | **Table 1. ADID comparisons.** The following table compares patients by ADID on a variety of variables in the study cohort. Each group is represented. Continuous variables are medians with 25^th^ and 75^th^ percentiles in parentheses aside from age, which is mean with standard deviation. Categorical variables are totals with percentage of the cohort in parentheses. H represents the test statistic for the Kruskal-Wallis test on non-normally distributed continuous variables. Confidence intervals for group differences are provided where relevant.  Median follow-up (H=7.6; df=3) was not significantly different by ADID (p>0.05). |
| Outcome data | 15* | *Cohort study*—Report numbers of outcome events or summary measures over time | 13 | **Table 1. ADID comparisons.** The following table compares patients by ADID on a variety of variables in the study cohort. Each group is represented. Continuous variables are medians with 25^th^ and 75^th^ percentiles in parentheses aside from age, which is mean with standard deviation. Categorical variables are totals with percentage of the cohort in parentheses. H represents the test statistic for the Kruskal-Wallis test on non-normally distributed continuous variables. Confidence intervals for group differences are provided where relevant. |
|  |  | *Case-control study—*Report numbers in each exposure category, or summary measures of exposure |  |  |
|  |  | *Cross-sectional study—*Report numbers of outcome events or summary measures |  |  |
| Main results | 16 | (*a*) Give unadjusted estimates and, if applicable, confounder-adjusted estimates and their precision (eg, 95% confidence interval). Make clear which confounders were adjusted for and why they were included | 6, 16, 17 | On multivariable logistic regression, when controlling for sex, CCI, surgical indication, and urinary diversion, ADID was not associated with likelihood of developing an in-house complication (p>0.05). An Indiana Pouch was associated with a greater likelihood of incurring a postoperative complication after BC [Odds ratio 4.3; Confidence Interval (CI) 1.5, 11.9; p<0.05]. On multivariable logistic regression, when controlling for sex, CCI, surgical indication, and urinary diversion, ADID was not associated with likelihood of 90-day readmission to the hospital. Both Indiana Pouch (Odds ratio 4.1; CI 1.3, 13.1; p<0.05) and a neobladder (Odds ratio 3.7; CI 1.4, 9.8; p<0.05) were associated with a greater likelihood of readmission within 90-days of discharge. On log-rank test for Kaplan-Meier survival analysis when comparing 90-day readmission after BC by ADID, no significant difference was appreciated (Figure 2; p>0.05). **Table 2. ADID multivariable regression model.** The following table is a regression model with an in-house complication after BC as the outcome. Significance is the p-value, OR is the adjusted odds ratio, and 95% CI is the confidence intervals for each variable. Any variables not displayed in the model can be assumed to not carry statistical significance or carried collinearity with other model variables. For ADID, D4 is the referent group, for urinary diversion ileal conduit is the referent group, and for indication “IC/BPS” is the referent group. **Table 3. ADID multivariable regression model.** The following table is a regression model with 90-day readmissions after BC as the outcome. B is the unadjusted odds ratio, S.E. is standard error, significance is the p-value, Exp(B) is the adjusted odds ratio, and 95% CI is the confidence intervals for each variable. Any variables not displayed in the model can be assumed to not carry statistical significance or carried collinearity with other model variables. . For ADID, D4 is the referent group, for urinary diversion ileal conduit is the referent group, and for indication “IC/BPS” is the referent group. |
|  |  | (*b*) Report category boundaries when continuous variables were categorized | N/a |  |
|  |  | (*c*) If relevant, consider translating estimates of relative risk into absolute risk for a meaningful time period | N/a |  |

Continued on next page

| Other analyses | 17 | Report other analyses done—eg analyses of subgroups and interactions, and sensitivity analyses | 6 | Sensitivity analyses modeling ADI as both a continuous and ordinal variable yielded consistent results, with ADI remaining non-significant and no meaningful changes in the direction or magnitude of effect estimates. These findings support the robustness of the primary model.  In the complication model, the p-value using ADID is 0.57; if ADI is modeled continuously, the p-value becomes 0.23, and, if the categories are modeled ordinally, the p-value is 0.30.  Similarly, in the readmission model, the categorized ADID p-value is 0.57, the continuous p-value is 0.58, and the ordinal p-value is 0.75. |
| --- | --- | --- | --- | --- |
| Discussion | | | | |
| Key results | 18 | Summarise key results with reference to study objectives | 6 | This large retrospective analysis of BC attempted to identify the impact of SES on peri/postoperative outcomes by using ADI scores and is the newest paper after updating our data in a series of BC that our research group has published on prior. No relevant associations were identified based on ADID. |
| Limitations | 19 | Discuss limitations of the study, taking into account sources of potential bias or imprecision. Discuss both direction and magnitude of any potential bias | 8 | There are multiple limitations in this study that require identification. First, this is a retrospective review, and subject to the inherent limitations that come with medical record chart reviews like selection bias and uncertainty regarding documentation. Additionally, a single-center analysis limits the external validity of the study findings. While utilizing ADI as a measure of SES has been validated, it is not the best measurement of an individual’s SES and is better for group SES, and this paper opens itself up to the possibility of ecological fallacy. Furthermore, our cohort was large for BC, which is a relatively uncommon procedure, but 183 patients are still a small sample size to analyze. All procedures were performed in an open fashion based on the surgeons’ comfort in this study, and it is possible that this influenced complication rates/the types of complications patients’ experienced. The regression models would be better improved by including calendar year, surgeon, indication severity, insurance status, distance from hospital, rurality, race and ethnicity, smoking, body mass index, functional status, frailty, prior radiation, prior pelvic surgery, chronic infection, chronic pain diagnosis, and preoperative opioid use if available. Unfortunately, due to a significant portion of missing variables/unavailable information in the patient record on the cohort studied, we elected to exclude these variables from the models. We also did not have equal proportions of patients in each ADID, specifically D4 had the least number of patients. With a greater share of D4, it is possible results could have differed, particularly if patients of the lowest SES drive potential differences. Regarding statistical power, the present study may be underpowered to detect modest but clinically meaningful differences between ADI groups. For example, observed differences in complication rates across groups may be clinically relevant despite not reaching statistical significance, and confidence intervals were wide, reflecting imprecision in these estimates. |
| Interpretation | 20 | Give a cautious overall interpretation of results considering objectives, limitations, multiplicity of analyses, results from similar studies, and other relevant evidence | 8 | BC carries a high morbidity rate postoperatively and healthcare utilization is high after surgery. This pattern held true, irrespective of patient ADID. These results provide some evidence that contradicts previous beliefs that patients of lower SES are the primary drivers of resource utilization in the healthcare system after BC. Nevertheless, this study is exploratory, and hypothesis generating in nature and should be viewed in the context of its multiple limitations. These results call for prospective validation. |
| Generalisability | 21 | Discuss the generalisability (external validity) of the study results | 8 | Additionally, a single-center analysis limits the external validity of the study findings. |
| Other information | |  | | |
| Funding | 22 | Give the source of funding and the role of the funders for the present study and, if applicable, for the original study on which the present article is based | 9 | Source(s) of Support and Funding-None |

*Give information separately for cases and controls in case-control studies and, if applicable, for exposed and unexposed groups in cohort and cross-sectional studies.

**Note:** An Explanation and Elaboration article discusses each checklist item and gives methodological background and published examples of transparent reporting. The STROBE checklist is best used in conjunction with this article (freely available on the Web sites of PLoS Medicine at http://www.plosmedicine.org/, Annals of Internal Medicine at http://www.annals.org/, and Epidemiology at http://www.epidem.com/). Information on the STROBE Initiative is available at www.strobe-statement.org.
